# The Difference in Translaminar Pressure Gradient and Neuroretinal Rim Area in Glaucoma and Healthy Subjects

**DOI:** 10.1155/2014/937360

**Published:** 2014-04-30

**Authors:** Lina Siaudvytyte, Ingrida Januleviciene, Arminas Ragauskas, Laimonas Bartusis, Indre Meiliuniene, Brent Siesky, Alon Harris

**Affiliations:** ^1^Eye Clinic, Lithuanian University of Health Sciences, Eiveniu Street 2, 51368 Kaunas, Lithuania; ^2^Telematics Science Laboratory of Kaunas University of Technology, Student**ų** Street 50, 51368 Kaunas, Lithuania; ^3^Glaucoma Research and Diagnostic Center, Eugene and Marilyn Glick Eye Institute, Indiana University School of Medicine, Indianapolis, IN 46202, USA

## Abstract

*Purpose*. To assess differences in translaminar pressure gradient (TPG) and neuroretinal rim area (NRA) in patients with normal tension glaucoma (NTG), high tension glaucoma (HTG), and healthy controls. *Methods*. 27 patients with NTG, HTG, and healthy controls were included in the prospective pilot study (each group consisted of 9 patients). Intraocular pressure (IOP), intracranial pressure (ICP), and confocal laser scanning tomography were assessed. TPG was calculated as the difference of IOP minus ICP. ICP was measured using noninvasive two-depth transcranial Doppler device. The level of significance *P* < 0.05 was considered significant. *Results*. NTG patients had significantly lower IOP (13.7(1.6) mmHg), NRA (0.97(0.36) mm^2^), comparing with HTG and healthy subjects, *P* < 0.05. ICP was lower in NTG (7.4(2.7) mmHg), compared with HTG (8.9(1.9) mmHg) and healthy subjects (10.5(3.0) mmHg); however, the difference between groups was not statistically significant (*P* > 0.05). The difference between TPG for healthy (5.4(7.7) mmHg) and glaucomatous eyes (NTG 6.3(3.1) mmHg, HTG 15.7(7.7) mmHg) was statistically significant (*P* < 0.001). Higher TPG was correlated with decreased NRA (*r* = −0.83; *P* = 0.01) in the NTG group. *Conclusion*. Translaminar pressure gradient was higher in glaucoma patients. Reduction of NRA was related to higher TPG in NTG patients. Further prospective studies are warranted to investigate the involvement of TPG in glaucoma management.

## 1. Introduction

Glaucoma is a progressive optic neuropathy leading to the retinal ganglion cell death and typical optic nerve head (ONH) damage [1]. It remains a disease with an unclear and complex underlying pathophysiology. Intraocular pressure (IOP) is the main and only modifiable risk factor for glaucoma [2]. Although lowering IOP helps to decelerate or stabilize the disease, a vast number of patients still show signs of glaucoma despite an IOP within normal range. Clearly other pathogenetic mechanisms beyond IOP are involved in the pathogenesis of glaucoma for certain individuals. Non-IOP factors such as lower systolic ocular perfusion pressure (OPP), reduced ocular blood flow, cardiovascular disease, and low systolic blood pressure (BP) have been identified as risk factors for primary open-angle glaucoma (POAG) [3–6]. Evidence shows that non-IOP factors can impact the apoptotic process associated with glaucoma [7].

Recently, researchers have emphasized not only IOP or vascular dysregulation, but also intracranial pressure's (ICP) role in glaucoma [8, 9]. The optic nerve is exposed not only to IOP in the eye, but also to ICP as it is surrounded by cerebrospinal fluid (CSF) in the subarachnoid space. Because the lamina cribrosa separates these two pressurized regions [10], the decrease in pressure that occurs across the lamina cribrosa (IOP-ICP) is known as the translaminar pressure gradient (TPG). A higher TPG may lead to abnormal function and optic nerve damage due to changes in axonal transportation, deformation of the lamina cribrosa, altered blood flow, or a combination thereof leading to glaucomatous damage. Besides, TPG may be the primarily pressure-related parameter for glaucoma [11–14], since the ONH is located at the junction between the intraocular space and the orbital retrobulbar space.

However, the role of TPG still remains unknown, because only invasive ICP measurements are available within the contemporary medicine (lumbar puncture or punction of brain ventricles—for patients with severe brain injury). The ideas for noninvasive ICP measurement have been appearing since about 1980. Numerous methods for finding the objects or physiological characteristics of cerebrospinal system that would be related to the ICP and its monitoring have been sought by many authors. Most of the proposed technologies were based on ultrasound and were capable of monitoring blood flow in intracranial or intraocular vessels, cranium diameter, or acoustic properties of the cranium [15]. Broad research has extended into sonography of optic nerve sheath and its relation with elevated ICP [16]. However, most of these correlation-based methods had the same problem—the need of individual patient specific calibration. Seeking to measure absolute ICP values, researchers from Kaunas University of Technology created noninvasive method, which does not need a patient specific calibration [17, 18]. The method is based on direct comparison of ICP value with the value of pressure Pe that is externally applied to the tissues surrounding the eyeball. Intracranial segment of ophthalmic artery (OA) is used as a natural sensor of ICP and extracranial segment of OA is used as a sensor of Pe. Special two-depth transcranial Doppler (TCD) device [17, 18] is used as a pressure balance indicator when ICP = Pe. Accuracy, precision, sensitivity, specificity, and diagnostic value of this method was proven with healthy subjects and patients with neurological diseases. This device has not yet been used in clinical studies to investigate TPG significance in glaucoma.

The aim of our study was to assess differences in TPG and NRA in patients with NTG, HTG, and healthy controls.

## 2. Materials and Methods

27 patients with NTG (age 56.6 (10.4)), HTG (age 54.7 (15.6)), and healthy controls (age 51.9 (6.6)) attended prospective pilot clinical study. Kaunas Regional Biomedical Research Ethics Committee approved the study protocol and all participants provided written informed consent, according to the Declaration of Helsinki. Indiana University School of Medicine, Department of Ophthalmology, provided services as the analysis reading center site for RBF images. Study objectives and methods were explained to all patients prior to examination. All patients signed an informed consent. Only one eye per patient was included in the study. The eye with greater glaucomatous damage was selected in the glaucoma patients. In healthy individuals, the eye was selected randomly.

Measurements included arterial BP, best-corrected visual acuity (BCVA), Goldmann applanation tonometry, confocal laser scanning tomography for optic nerve disc structural changes (HRT, Heidelberg Retina Tomograph, Heidelberg Engineering, Heidelberg, Germany), and noninvasive ICP (Vittamed 205, Kaunas, Lithuania). TPG was calculated as the difference of IOP minus ICP.

Noninvasive absolute ICP value was measured using two-depth TCD device that does not need an individual patient specific calibration and is based on simultaneous monitoring of blood flow velocity pulsations in intracranial and extracranial OA segments. The value of external pressure, when OA blood flow parameters in both segments are equal, was fixed and expressed automatically in absolute units mmHg. A head frame with fixed ultrasound transducer was added to close eyelid of a patient. Patients remained in the supine position. Special acoustic conducted gel was used for a better ultrasonic contact. An external pressure Pe has been produced by a small ring cuff placed over the tissues surrounding the eyeball. Pe was automatically increased gradually from 0 to 20 mmHg by pressure steps equal to 4 mmHg. In order to decrease ICP value sampling error the measurement was repeated with external pressure Pe increased by 2 mmHg pressure steps until 12 mmHg, if first measured absolute ICP value was lower than 10 mmHg (Figure 2). The duration of the procedure was up to 10 minutes.

The inclusion criteria were NTG/HTG patients over 18 years of age with diurnal IOP lower/higher than 21 mmHg, and willing to sign informed consent form prior to initiation of the study. Pregnant or nursing women, patients with uncontrolled systemic diseases, and patients with a history of allergy to local anesthetics, orbital/ocular trauma, or other diseases that could influence study results were excluded from the study. Patients were included in the HTG group if they were diagnosed with POAG by a glaucoma specialist and had characteristic OND changes, visual field loss consistent with glaucoma, and IOP higher than 21 mmHg. Patients were considered to have NTG if NTG was diagnosed by a glaucoma specialist and had characteristic OND changes, visual field loss consistent with glaucoma, and IOP always less than 21 mmHg before treatment. Current medical treatment, including topical IOP lowering drugs, was continued. The control group was composed of healthy adult volunteers with no history of glaucoma or other diseases that could bias the results.

Statistical analysis was performed using computer program SPSS 17.0 for Windows. All variables were defined by methods of descriptive statistics. The analysis of the quantitative variables included calculation of the mean and standard deviation (×(SD)). The hypothesis of equality among three groups was analysed using the Kruskall-Wallis test. Association between categorical variables or abnormally distributed continuous variables was assessed by Spearman's correlation. The level of significance *P* < 0.05 was considered significant.

## 3. Results

27 patients (74.1% women, 25.9% men) with NTG, HTG, and healthy controls were included in the study (each group consisted of 9 patients). The study groups did not vary significantly in age (*P* > 0.05). Patients' characteristics are provided in Table 1.

Changes in IOP, ICP, TPG, and optic nerve disc structure are shown in Table 2. NTG patients had significantly lower IOP (13.7 (1.6) mmHg), NRA (0.97 (0.36) mm^2^), and retinal nerve fiber layer thickness (0.15 (0.07) mm), comparing with HTG and healthy patients, *P* < 0.05. ICP was lower in NTG (7.4 (2.7) mmHg), compared with HTG (8.9 (1.9) mmHg) and healthy subjects (10.5 (3.0) mmHg); however the difference between groups was not statistically significant (*P* > 0.05). The difference between TPG for healthy (5.4 (7.7) mmHg) and glaucomatous eyes (NTG 6.3 (3.1) mmHg, HTG 15.7 (7.7) mmHg) was statistically significant (*P* < 0.001).

**Figure 1:**
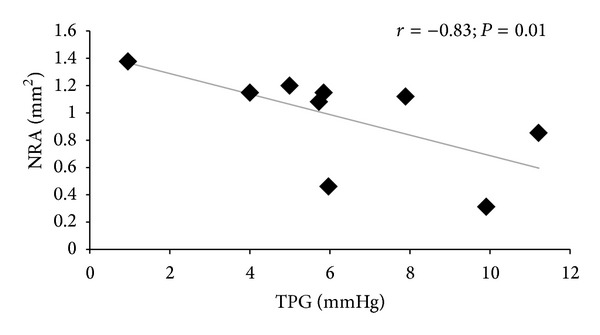
Correlation between translaminar pressure gradient and neuroretinal rim area in normal tension glaucoma patients. ∗Spearman's correlation. Significance level *P* < 0.05. *r*: correlation coefficient, TPG: translaminar pressure gradient, NRA: neuroretinal rim area.

**Figure 2:**
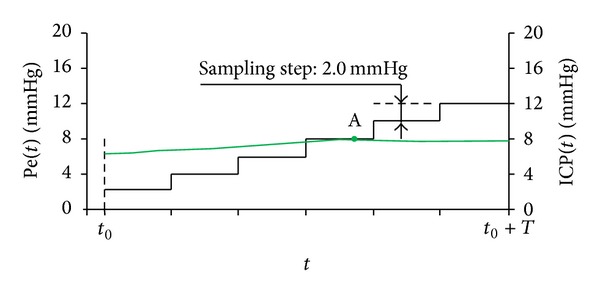
Noninvasive intracranial pressure absolute value measurement procedure. Time diagram of the externally applied pressure Pe(*t*) when pressure step is equal to 2 mmHg (solid black step function) and intracranial pressure ICP(*t*) (thin green line). Point (A) is pressure balance Pe = aICP point when pressure step is equal to 2 mmHg, respectively.

Correlations between NRA and IOP, ICP, and TPG in glaucoma patients and healthy subjects are shown in Table 3. A negative correlation between TPG and NRA (*r* = −0.83; *P* = 0.01) was observed in the NTG group (Figure 1), while no such relation was identified in the other groups. We also found that lower ICP was related to lower diastolic BP in the NTG group (*r* = 0.81; *P* = 0.001).

## 4. Discussion

A pressure imbalance between the two circulating fluids of the nervous system may be the cause of glaucomatous damage to the optic nerve. Jonas and Budde showed that the optic disc appearance in NTG patients could be remarkably similar to the optic nerve head morphology in HTG patients [19]. Recent studies supported the hypothesis that an abnormally low ICP can lead to glaucomatous optic nerve damage [10–12, 14, 20–23]. Berdahl et al. in retrospective analysis of patients who had a lumbar puncture revealed a significantly lower lumbar CSF pressure among individuals with NTG than those with HTG or healthy subjects. Further, they reported that the amount of glaucomatous damage to the optic nerve correlated with the difference in IOP and lumbar CSF pressure [22]. More recent prospective study compared CSF pressures in a cohort of patients with POAG to those of a control group slated for lumbar puncture for other reasons. The results were very similar to those in the retrospective studies, with the control group having the highest CSF pressure and the smallest TPG [23]. The findings of our study agree with previous investigations. In our study we found that ICP was 3-2 mmHg lower in patients with open-angle glaucoma, especially in NTG, compared with healthy subjects, while TPG was higher in HTG and NTG patients, compared with healthy subjects. However, the correlation between NRA and TPG was found just in NTG group. It confirmed the idea that decreased ICP could result in an increased TPG and lead to glaucomatous damage.

In prospective study, Ren and colleagues found that in normal subjects CSF pressure is related to the systemic arterial BP and the IOP [23]. According to several population-based studies, IOP is also related to the systemic arterial BP so that the pressures in all three fluid filled compartments are related to each other [24, 25]. In our study we found positive correlation between ICP and diastolic BP in NTG, while no such relation was identified in other groups. This data suggests that diastolic BP may be an important consideration in NTG management.

There are several limitations to acknowledge in our study. First, the number of patients in NTG, HTG, and control groups was small and therefore our data should be considered as a pilot study. Second, this study did not include a washout period and hypotensive agents could have possible effects on ICP, especially carbonic anhydrase inhibitors. Third, ICP was measured in the supine position, while IOP was assessed in the sitting position; therefore our calculated TPD is only a surrogate for the actual TPG. Fourth, it has remained unclear whether the lumbar CSF pressure is directly related to the CSF pressure in the orbit around the optic nerve. It is known that TPG is calculated as the difference of IOP minus orbital CSF pressure. Usually lumbar CSF pressure is used as surrogate for the orbital CSF pressure. It is also known that CSF column height measurement is the “gold standard” invasive ICP evaluation method. We used a novel noninvasive ICP measurement method in which sensitivity, specificity, and diagnostic value were proven with healthy subjects and patients with neurological diseases [17, 18].

## 5. Conclusion

Translaminar pressure gradient was higher in glaucoma patients than in healthy controls. Reduction of neuroretinal rim area was related to higher TPG in NTG patients. Further prospective studies are warranted to investigate the involvement of TPG in glaucoma management.

## Figures and Tables

**Table 1:** Patients characteristics.

	NTG group (*N* = 9) Mean (SD)	HTG group (*N* = 9) Mean (SD)	Control group (*N* = 9) Mean (SD)	*P* level
Sex (*N* (%))				0.46
Male	3 (33.3%)	3 (33.3%)	1 (11.1%)	
Female	6 (66.7%)	6 (66.7%)	8 (88.9%)	
Age (years)	56.6 (10.4)	54.7 (15.6)	51.9 (6.6)	0.7
Range	41–72	36–88	42–60	
Best-corrected visual acuity	0.98 (0.04)	0.68 (0.41)	0.97 (0.1)	0.03*
Systolic blood pressure (mmHg)	142.1 (16.6)	156.4 (23.0)	132.2 (6.4)	0.02*
Diastolic blood pressure (mmHg)	86.0 (9.7)	90.9 (9.4)	85.7 (4.6)	0.35

*Kruskall-Wallis test. **Chi-square test. Significance level *P* < 0.05. NTG: normal tension glaucoma; HTG: high tension glaucoma; SD: standard deviation; *N*: number.

**Table 2:** Changes in intraocular pressure, intracranial pressure, translaminar pressure gradient, and optic nerve disc structure.

	NTG group (*N* = 9) Mean (SD)	HTG group (*N* = 9) Mean (SD)	Control group (*N* = 9) Mean (SD)	*P* level
IOP (mmHg)	13.7 (1.6)	24.7 (6.8)	15.9 (2.1)	<0.001*
ICP (mmHg)	7.4 (2.7)	8.9 (1.9)	10.5 (3.0)	0.06
TPG (mmHg)	6.3 (3.1)	15.7 (7.7)	5.4 (3.3)	<0.001*
Disc area (mm^2^)	2.03 (0.5)	1.96 (0.4)	2.1 (0.2)	0.68
NRA (mm^2^)	0.97 (0.36)	1.32 (0.67)	1.79 (0.20)	0.003*
Linear cup/disc ratio	0.70 (0.14)	0.52 (0.27)	0.38 (0.08)	0.002*
RNFL thickness (mm)	0.15 (0.07)	0.20 (0.08)	0.28 (0.02)	0.001*

*Kruskall-Wallis test. Significance level *P* < 0.05. NTG: normal tension glaucoma; HTG: high tension glaucoma; SD: standard deviation; *N*: number; IOP: intraocular pressure; ICP: intracranial pressure; TPG: translaminar pressure gradient; NRA: neuroretinal rim area; RNFL: retinal nerve fiber layer.

**Table 3:** Correlations between neuroretinal rim area and intraocular pressure, intracranial pressure, and translaminar pressure gradient in glaucoma patients and healthy subjects.

	NTG group (*N* = 9)		HTG group (*N* = 9)		Control group (*N* = 9)	
	*r*	*P* level	*r*	*P* level	*r*	*P* level
NRA correlated with						
IOP	−0.25	0.51	−0.20	0.60	−0.03	0.95
ICP	0.80	0.01*	−0.34	0.37	0.06	0.88
TPG	−0.83	0.01*	0.21	0.59	−0.22	0.57

*Spearman's correlation. Significance level *P* < 0.05. *r*: correlation coefficient; NTG: normal tension glaucoma; HTG: high tension glaucoma; *N*: number; IOP: intraocular pressure; ICP: intracranial pressure; TPG: translaminar pressure gradient; NRA: neuroretinal rim area.
